# Pre-purification of diatom pigment protein complexes provides insight into the heterogeneity of FCP complexes

**DOI:** 10.1186/s12870-020-02668-x

**Published:** 2020-10-06

**Authors:** Marcel Kansy, Daniela Volke, Line Sturm, Christian Wilhelm, Ralf Hoffmann, Reimund Goss

**Affiliations:** 1 Institute of Biology, Leipzig University, Johannisallee 21-23, 04103 Leipzig, Germany; 2 Institute for Bioanalytical Chemistry, Centre for Biotechnology and Biomedicine, Leipzig University, Deutscher Platz 5, 04103 Leipzig, Germany; 3 Institute of Biology, Leipzig University, Permoserstraße 15, 04318 Leipzig, Germany

**Keywords:** Anion exchange chromatography, Fucoxanthin chlorophyll protein, Lhcx, Mass spectrometry, Photosystem I, Photosystem II

## Abstract

**Background:**

Although our knowledge about diatom photosynthesis has made huge progress over the last years, many aspects about their photosynthetic apparatus are still enigmatic. According to published data, the spatial organization as well as the biochemical composition of diatom thylakoid membranes is significantly different from that of higher plants.

**Results:**

In this study the pigment protein complexes of the diatom *Thalassiosira pseudonana* were isolated by anion exchange chromatography. A step gradient was used for the elution process, yielding five well-separated pigment protein fractions which were characterized in detail. The isolation of photosystem (PS) core complex fractions, which contained fucoxanthin chlorophyll proteins (FCPs), enabled the differentiation between different FCP complexes: FCP complexes which were more closely associated with the PSI and PSII core complexes and FCP complexes which built-up the peripheral antenna. Analysis by mass spectrometry showed that the FCP complexes associated with the PSI and PSII core complexes contained various Lhcf proteins, including Lhcf1, Lhcf2, Lhcf4, Lhcf5, Lhcf6, Lhcf8 and Lhcf9 proteins, while the peripheral FCP complexes were exclusively composed of Lhcf8 and Lhcf9. Lhcr proteins, namely Lhcr1, Lhcr3 and Lhcr14, were identified in fractions containing subunits of the PSI core complex. Lhcx1, Lhcx2 and Lhcx5 proteins co-eluted with PSII protein subunits. The first fraction contained an additional Lhcx protein, Lhcx6_1, and was furthermore characterized by high concentrations of photoprotective xanthophyll cycle pigments.

**Conclusion:**

The results of the present study corroborate existing data, like the observation of a PSI-specific antenna complex in diatoms composed of Lhcr proteins. They complement other data, like e.g. on the protein composition of the 21 kDa FCP band or the Lhcf composition of FCPa and FCPb complexes. They also provide interesting new information, like the presence of the enzyme diadinoxanthin de-epoxidase in the Lhcx-containing PSII fraction, which might be relevant for the process of non-photochemical quenching. Finally, the high negative charge of the main FCP fraction may play a role in the organization and structure of the native diatom thylakoid membrane. Thus, the results present an important contribution to our understanding of the complex nature of the diatom antenna system.

## Background

The photosynthetic pigment protein complexes comprising the photosystem (PS) II and PSI core complexes with their specific light-harvesting complexes (LHC) are embedded into the thylakoid membrane. In contrast to higher plants, where the thylakoid membrane system is differentiated into grana and stroma membranes [7], the thylakoids of diatoms are usually arranged as regular stacks of three membranes [29]. Despite the regular arrangement recent results have proposed that a heterogeneous distribution of PSII and PSI, as it exists in the grana and stroma membranes, is also present in the diatom thylakoid membranes. According to the model of Lepetit et al. [22] PSI with its specific FCP complex is mainly located in the peripheral membrane regions together with an enrichment of the negatively charged membrane lipid sulfoquinovosyldiacylglycerol (SQDG). The inner membrane regions are preferentially occupied by PSII and the PSII-specific FCP complexes which are surrounded by lipid phases enriched with the neutral galactolipid monogalactosyldiacylglycerol (MGDG). Further evidence for the spatial separation of PSII and PSI stems from the work of Bina et al. [4] who showed that the thylakoid membranes of the pennate diatom *P. tricornutum* contain large areas which are exclusively occupied by a supercomplex of PSI with its associated antenna composed of Lhcr proteins when the algae are cultivated with red light of a low light intensity. Flori et al. [10], using a combination of biochemical, structural and physiological data were able to show that the three-dimensional network of the thylakoid membrane of *P. tricornutum* is far more complex than the simple layout of three loosely connected membranes. In addition, the authors found evidence for a compartmentalization of the two photosystems. In accordance with the model of Lepetit et al. [22] they propose that PSII is located in the core membranes whereas PSI is enriched in the peripheral, stroma-facing membranes.

The light-harvesting antenna system of diatoms is assembled from membrane intrinsic FCP proteins. Due to their distribution within the thylakoid membrane and their specific functions these proteins are divided into three classes, termed Lhcf, Lhcr and Lhcx proteins. The Lhcf proteins constitute a major light-harvesting antenna, the so called peripheral FCP complex [13, 15, 23, 27], which supplies both photosystems with excitation energy. The Lhcr proteins are preferentially associated with PSI and form PSI-specific antenna complexes [18, 33]. The Lhcx proteins are supposed to play an important role in the process of non-photochemical quenching (NPQ) of chlorophyll (Chl) a fluorescence [2], an essential photoprotection mechanism in photosynthetic organisms [11]. In diatoms Lhcx proteins are only found in substoichiometric ratios in comparison to Lhcf proteins [16], and expression of some of these genes was shown to be induced upon high light or temperature stress [36]. In the centric diatom *T. pseudonana;* at least 30 FCP proteins where found [1], of which only six belong to the Lhcx family. This is in line with their role in the regulation of photoprotection, a function which is also observed in the pennate diatom *P. tricornutum* [31, 32]. As it was demonstrated for the Lhcx proteins, the Lhcf composition of the diatom antenna system depends on the light intensity during cultivation ([12, 13] for *T. pseudonana* [15, 16] for *P. tricornutum* and *C. meneghiniana*, respectively).

The basic structure of the different FCP proteins within the native thylakoid membrane is the FCP trimer which can be found in both the pennate and centric diatoms [13, 15, 16, 24]. This unit has been termed FCPa, and a detailed analysis following the subfractionation of FCP complexes revealed various trimeric subtypes which differ in their stochiometric and even individual Lhcf composition [15, 16]. In *C. meneghiniana* four subtypes were recently described and termed FCPa1–4, with Lhcf1 being the main subunit in FCPa1, FCPa3 and FCPa4, whereas Lhcf4/Lhcf6 is enriched in the FCPa2 trimer [16]. In the native membrane it seems that specific trimers also form higher oligomeric structures [5]. These hexamers or nonamers build the peripheral antenna system and have been termed FCPb or FCPo (FCP in oligomeric state). In the centric diatom *C. meneghiniana* the two oligomeric subtypes FCPb1 and FCPb2 were described [16], with Lhcf3 dominating both antenna complexes. However, as shown for the pennate diatom *P. tricornutum* [24] and the centric *C. meneghiniana* [23], these oligomeric structures are sensitive to the solubilization conditions, i.e. to the type and concentration of the used detergent. Lepetit and coworkers were only able to retain the FCPo structure by decreasing the detergent (n-dodecyl β-D-maltoside (β-DM)) concentration from 2 to 0.5%. Interestingly, it seems that these oligomeric structures are more resistant to the solubilization conditions in centric diatoms. Employing clear-native electrophoresis following a solubilization with a detergent concentration of 4%, Nagao et al. [27] were able to detect FCPo structures in three different centric diatoms, whereas no FCPo structure could be observed in the pennate *P. tricornutum.* The importance of the solubilization conditions for the preservation of native pigment-protein complex structures was also demonstrated recently by Calvaruso et al. [6]. With the help of a very mild treatment with the detergent α-DM, the purification of various photosystem-antenna supercomplexes of *T. pseudonana* was possible. According to these results, the photoprotective Lhcx6_1 protein was found in conjunction with PSII, whereas Lhcr proteins where preferentially associated with PSI. Furthermore, the authors identified Lhcf8/9 as the main antenna protein in the peripheral FCP antenna system. With respect to the concept of the FCP trimer as the basic unit of diatom antenna proteins, this idea was recently challenged by the first detailed cryo-electron microscopic and X-ray crystallography studies on diatom pigment-protein complexes. Using cryo-electron microscopy Wang et al. [35] resolved a PSII-antenna supercomplex from the centric diatom *Chaetoceros gracilis* and reported a tetrameric organization of FCP proteins in the vicinity of PSII. With regard to the molecular structure and stoichiometric organization of individual FCP proteins, x-ray crystallography data of the Lhcf3 and Lhcf4 proteins from *P. tricornutum* at 1.8 Å reveal a dimeric organization [34].

It is the intention of this study to present a rapid and reproducible method for the pre-purification of native pigment protein complexes of the thylakoid membrane of the centric diatom *T. pseudonana*. The method employs anion exchange chromatography (AEC) for the separation of FCP complexes but additionally allows the pre-purification of PSII and PSI core complexes. The pre-purified PSII and PSI core complexes can serve as starting material for further purification steps. More important, however, is the observation that the present purification method preserves some of the interactions between the FCP complexes and the core complexes of the photosystems. It thus allows to differentiate between FCP proteins which are rather tightly associated with either the PSI or PSII core complexes and FCP proteins which are only loosely connected and build-up the peripheral main light-harvesting complexes of *T. pseudonana*. In the present study the separated AEC fractions were characterized by spectroscopic means and their pigmentation was determined by HPLC. Finally, the protein composition was analysed by mass spectrometry with a special emphasis on the Lhcf, Lhcr and Lhcx protein composition of the different FCP sub-populations.

## Results

### Separation of pigment protein complexes by AEC

**Fig. 1:**
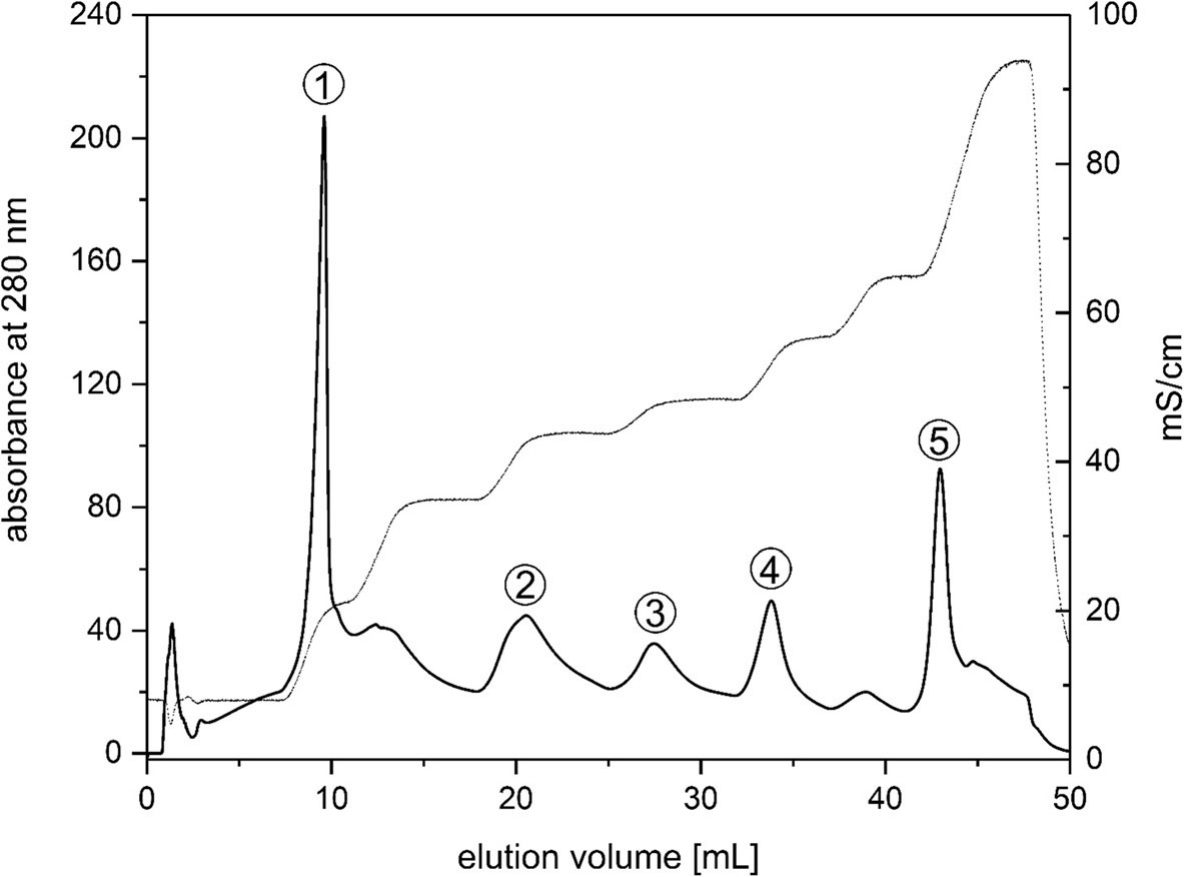
Elution profile of the pigment protein complexes of *T. pseudonana* separated by anion exchange chromatography (AEC). Figure 1 depicts the protein absorption at 280 nm and the stepwise increase of the KCl concentration. Before the separation, isolated thylakoids were solubilized with a β-DM per Chl ratio of 20. Solubilized thylakoids with a total amount of 200 to 500 μg Chl were loaded onto the AEC column. The numbers of the peaks denote the fractions that were collected and further characterized. Figure 1 shows a typical elution profile. For more information see the Methods section

The solubilized *T. pseudonana* thylakoid membranes were separated by AEC in eight well resolved peaks (Fig. 1). The first peak consisted of solubilized material which eluted at the initial low salt concentrations. This putative free pigment fraction, also observed by Gundermann et al. [16], was not further analysed in the present study. The following five major and two minor peaks were detected after each step-wise increase of the salt concentration from 30 to 500 mM KCl. Increasing retention times indicated most likely a higher negative surface charge of the respective pigment proteins. For the following experiments the fractions corresponding to the five major peaks were pooled and termed Fractions 1 to 5 (Fig. 1). All fractions contained pigment protein complexes of the thylakoid membrane of *T. pseudonana* in different states of purity with different protein and pigment concentrations. While the absorbance at 280 nm of Fractions 1 and 5 indicates high protein and pigment concentrations, fractions 2, 3 and 4 presumably contain reduced protein and pigment quantities. Fractions 1 to 5 were further characterized by absorption and fluorescence spectroscopy, determination of their pigment content and analysis of their protein composition. The peak with a retention time between 10 and 15 mL was variable and depending on slight differences in the culture age and growth light conditions more or less pronounced. Thus, it was not investigated by mass spectrometry. However, the other measuring techniques employed in the present study provided evidence that it represented a PSII core complex fraction.

### Spectroscopic features of the separated pigment protein complexes

**Fig. 2:**
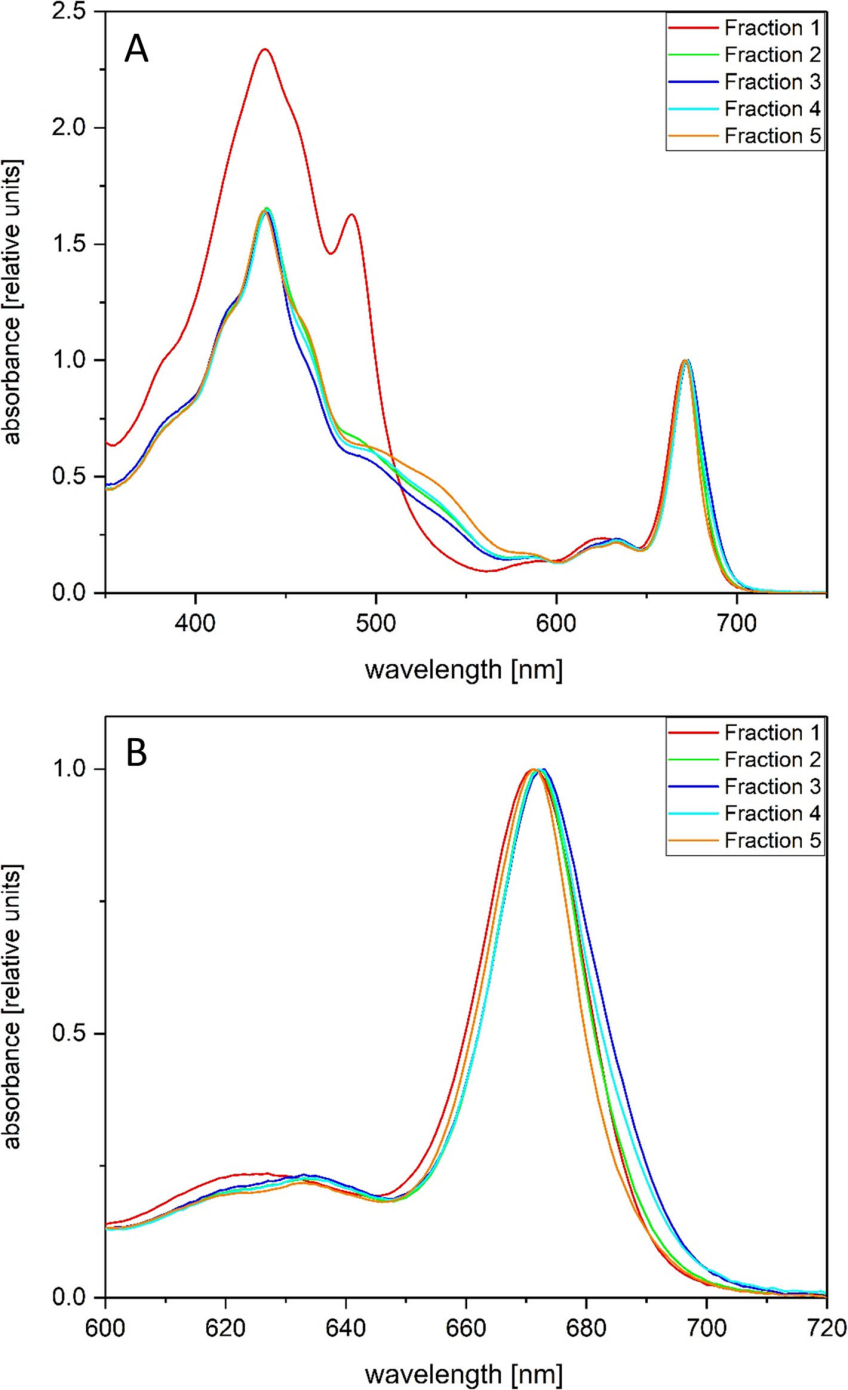
Absorption spectra of the different AEC fractions. The absorption spectra were normalized to the Q_Y_ band of Chl a. For the measurements the Chl concentration of the isolated pigment protein complexes was adjusted in such a way that the absorption in the blue part of the spectrum did not exceed absorption values of 1. Figure 2a shows the absorption spectrum in the wavelength range from 350 to 750 nm, Fig. 2b presents a detailed view of the red absorption maximum of Chl a. Figure 2 shows typical absorption spectra. For additional information see the Methods section

Fraction 1 (Fig. 1) was characterized by a relatively high absorption in the blue to blue-green region of the absorption spectrum (Fig. 2a). Prominent absorption maxima were observed at around 440 and 490 nm, accompanied by a shoulder at 460 nm. The maximum at 440 and the shoulder at 460 nm corresponded to the blue absorption maxima of Chl a and Chl c, respectively, the pronounced maximum at 490 nm was related to a strong carotenoid absorption in this wavelength region. It corresponded to the third absorption maximum of the absorption spectrum of carotenoid molecules, which typically shows three defined absorption bands in the blue to blue-green part of the spectrum. The first and second absorption bands of the carotenoid absorption spectrum were not visible as defined maxima since they were concealed by the Chl a and Chl c absorption bands. The high absorption in the blue to blue-green part of the spectrum, together with the pronounced carotenoid peak at around 490 nm, demonstrated that Fraction 1 was enriched in carotenoids. Since the main carotenoid of diatoms, i.e. fucoxanthin (Fx), is characterized by a rather broad, undefined absorption spectrum, the clear maximum at 490 nm indicates a strong contribution of the xanthophyll cycle pigment (diadinoxanthin) DD to the absorption of Fraction 1. The absorption spectra of Fractions 2 to 5 were dominated by Chl a absorption in the blue and red part of the spectrum. While the Soret band of Chl a (singulet state 2 transition) was located at around 440 nm, the Q_Y_ absorption band (singulet state 1 transition) was found at around 670 nm. Chl c was visible in the blue part of the spectrum as a shoulder at around 460 nm. Furthermore, and in contrast to Fraction 1, the absorption of protein bound Fx was clearly detected in the wavelength region from 490 to 550 nm. The Chl c shoulder and the Fx absorption were pronounced in Fraction 5, which indicated that the respective pigment protein complexes were enriched in these pigments. Further interesting observations could be derived from the data presented in Fig. 2b which depicts the red part of the absorption spectrum of the different AEC fractions in closer detail. It became obvious that the Q_Y_ absorption of Chl a, which was located at around 670 nm in Fractions 1, 2, and 5, was shifted towards longer wavelengths in Fractions 3 and 4. This indicated the presence of longer wavelength absorbing Chl a molecules in the pigment protein complexes isolated in Fractions 3 and 4.

**Fig. 3:**
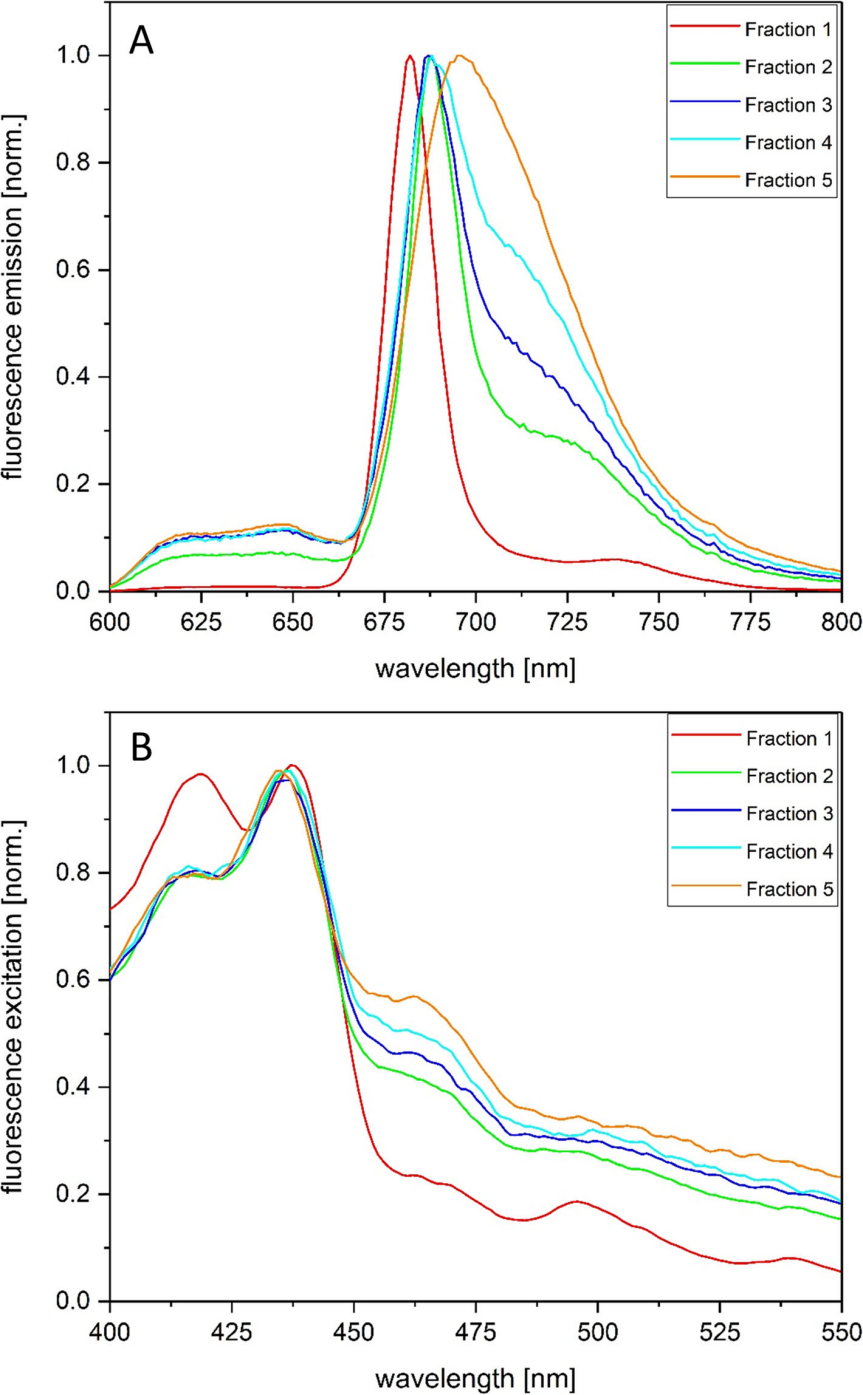
77 K fluorescence spectra of the five AEC fractions. The spectra were normalized to the fluorescence emission maximum (Fig. 3a) or the excitation maximum of the Chl a fluorescence (Fig. 3b). For the 77 K fluorescence measurements the pigment protein complexes were adjusted to an optical density of 0.1 in the red part of the spectrum and then diluted with glycerol until a final glycerol concentration of 60% was obtained. Figure 3a shows the fluorescence emission spectra with a constant excitation at 440 nm, for the excitation spectra depicted in Fig. 3b the constant emission wavelength was set to the maximum of the emission spectrum. Fig. 3 shows typical emission and excitation spectra. For further details see the Methods section

The 77 K fluorescence emission spectra of the AEC fractions showed differences after excitation with 440 nm light which corresponds to the Chl a absorption maximum in the blue part of the spectrum (Fig. 3a). Fraction 1 was characterized by a homogeneous peak shape and the shortest emission maximum at wavelengths of around 682 nm. The emission maxima of Fractions 2, 3 and 4 were shifted towards longer wavelengths and were typically found at around 687–688 nm. In contrast to Fraction 1, these fractions exhibited a pronounced fluorescence emission with increasing contributions in the wavelength range above 700 nm. This observation corresponds with the shift of the Q_Y_ absorption maximum of Chl a towards longer wavelengths in these fractions (Fig. 2b). The fluorescence emission spectra of Fraction 5 were variable and the spectra were sometimes dominated by shorter and sometimes by longer wavelength contributions (Fig. 3a, Additional file 8).

The fluorescence excitation spectra of the different fractions (Fig. 3b) showed interesting differences to the respective absorption spectra. Fraction 1, which was characterized by prominent carotenoid absorption bands, exhibited only a strong Chl a fluorescence emission at around 682 nm when Chl a was excited with blue light. Excitation of Chl c and carotenoid molecules with light above 450 nm only induced a weak Chl a fluorescence emission. This demonstrated that the carotenoids which were present in high amounts in Fraction 1, mainly DD according to the absorption spectrum, were not able to efficiently transfer excitation energy to Chl a. The peak at around 420 nm in the excitation spectrum of Fraction 1 may furthermore indicate the presence of pheophytin in this fraction. Fractions 2 to 5 showed Chl a fluorescence emission after excitation of Chl a, Chl c and Fx. Chl c excitation was visible as a maximum at around 460 nm, whereas Fx excitation could be attributed to the wavelength range from 490 to 550 nm. The most prominent Chl a fluorescence after excitation of Chl c and Fx was visible in Fraction 5 which corresponds well with the most pronounced Chl c and Fx absorption bands in this Fraction.

### Pigment composition of the pigment protein complexes

**Fig. 4:**
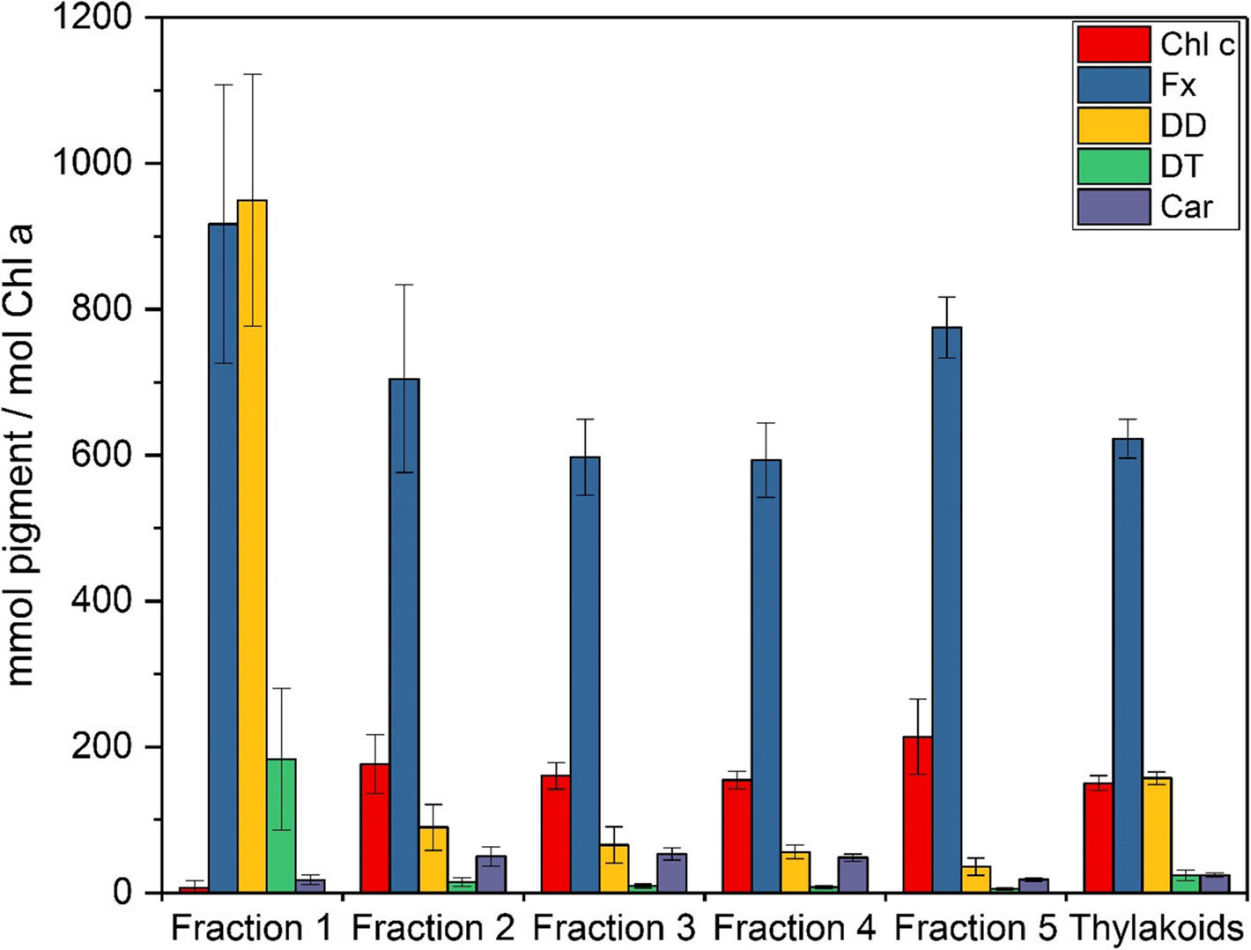
Pigment composition of the different AEC fractions and thylakoid membranes of *T. pseudonana*. The pigment composition is depicted as mM pigment M^− 1^ Chl a. Figure 4 shows the mean values of three independent measurements with the respective standard deviations. For further information see the Methods section

The pigment analysis of the different AEC fractions (Fig. 4 and Additional file 1) supports the findings derived from the spectroscopic measurements. Fraction 1 contained high amounts of the main light-harvesting xanthophyll Fx and the xanthophyll cycle pigment DD. Especially the high concentration of DD, which slightly exceeded the Fx concentration in this fraction, is noteworthy. The enrichment of xanthophyll cycle pigments in the first fraction was also documented by the significant concentration of diatoxanthin (Dt) which was present in these samples. Dt was present in all fractions because the *T. pseudonana* cells were harvested during the light period of the light/dark cycle used for algal cultivation before the preparation of the thylakoid membranes was performed. β-carotene, on the other hand, was observed in only low concentrations in Fraction 1. The presence of high amounts of DD supported the annotation of the 490 nm absorption peak in Fraction 1 to the third absorption maximum of DD (Fig. 2a). The concentration of the second Chl, Chl c, was even lower than that of β-carotene. Fractions 2 to 4 showed a comparable pigment composition. Besides Chl a, Fx was the main pigment in these fractions followed by Chl c and DD, Dt was present in low concentrations. Interestingly, the Fx concentration decreased slightly with increasing fraction number. In comparison to Fraction 1 β-carotene was present in higher concentrations in Fractions 2 to 4. The last fraction of the AEC separation, Fraction 5, contained high amounts of the typical diatom light-harvesting pigments Fx and Chl c. DD, Dt and β-carotene, on the other hand, were present in only low concentration. The pigment composition of Fractions 2 to 5 was in line with the absorption spectra of the respective fractions which, besides Chl a absorption, were dominated by Chl c and Fx absorption. The highest contribution of Chl c and Fx to the overall absorption spectrum was found for Fraction 5 which corresponds well with the highest Fx and Chl c concentration in this sample.

### Protein composition of the separated pigment protein complexes

**Fig. 5:**
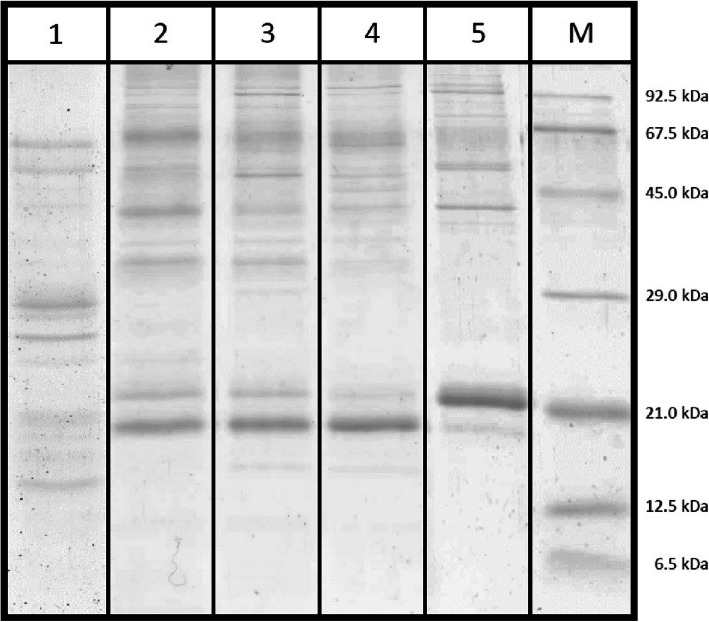
Representative gel image of the protein composition of the five AEC fractions determined by SDS-PAGE. Numbers 1 to 5 in Fig. 5 correspond to the respective fractions depicted in Fig. 1. Lanes 2 to 5 are derived from the original gel depicted in Additional file 7A, lane 1 is derived from the original gel shown in Additional file 7B. Proteins were stained with colloidal Coomassie Brilliant Blue. M denotes the molecular weight markers. For detailed information on the nature of the protein bands see section ‘Protein composition of the separated pigment protein complexes’. MS data for the 18 and 21 kDa FCP bands of lanes 2 to 5 (i.e. AEC fractions 2 to 5) are provided in Additional file 5. MS data for the complete analysis of photosynthetic proteins of fractions 1 to 4) can be found in Additional file 6

The FCP complexes of *T. pseudonana* were visible as prominent 21 and 18 kDa bands on the SDS-gels (Fig. 5 and Additional file 7A and B). Figure 5 shows representative SDS-gels; for all AEC separations protein analyses by SDS-PAGE were performed. Additional file 7A and B show SDS-gels of two separations and thus allow to judge the reproducibility of the AEC fractionation and the protein determination by SDS-PAGE. In Fraction 1 the 18 kDa FCP band was visible as weakly coloured band while the 21 kDa band could not be detected in the gel. Fractions 2 to 4 showed an increasing intensity of the lower molecular weight FCP band, whereas Fraction 5 was characterized by the almost single presence of the 21 kDa FCP band. In addition to the FCP bands further bands at higher apparent molecular weights were visible in different fractions. For Fraction 1, bands in the 25 to 30 kDa range were stained. Fractions 2 and 3 contained additional bands in the 30 to 35 kDa region, which indicate the presence of the PSII reaction centre proteins D1 and D2 and the 33 kDa (PsbO) protein of the oxygen evolving complex (OEC). Further bands were observed at around 45 kDa that may represent the inner antenna proteins CP43 and CP47. In Fractions 4 and 5 the bands in the 30 to 35 kDa range could not be detected. Bands visible above the 69 kDa protein marker in Fractions 2, 3 and 4 could represent either the PSI core proteins PsaA and PsaB or a D1/D2 heterodimer. These bands were prominent in Fractions 3 and 4 due to the absence of the PSII proteins. Fraction 5 was clearly dominated by bands that correspond to the FCP proteins. PSII proteins could not be detected in this fraction and the intensity of the protein bands corresponding to the PSI core proteins was significantly lower than for Fractions 3 and 4.

The proteins present in the 18 and 21 kDa bands seen in Fractions 2 to 5 were analysed by mass spectrometry (Mass spectrometry analysis 1, Table 1) considering only proteins identified by at least two confident and unique peptides and a protein score of ≥1000. All identified antenna and photosystem proteins are listed in Additional file 5. Since Fraction 1 showed only a weak 18 kDa band, while the 21 kDa band was completely missing, it was not analysed by Mass spectrometry analysis 1. However, the protein composition of Fraction 1 was determined by Mass spectrometry analysis 2 (see below). The 18 kDa band of Fraction 2 contained Lhcf1, Lhcf2, Lhcf4, Lhcf5, Lhcf6, Lhcf8 and Lhcf9, while only Lhcf1, Lhcf2, Lhcf5, and Lhcf6 were detected in the corresponding bands of Fraction 3 and Fraction 4. The 18 kDa band of Fraction 5 contained only Lhcf8 and Lhcf9. The 21 kDa band, representing the dominant FCP protein band of Fraction 5, contained Lhcf8 and Lhcf9, which was also true for all other fractions displaying the 21 kDa band. Other Lhc proteins were not identified in any 21 kDa band.

**Table 1:** Analysis of the 18 and 21 kDa FCP bands of the different AEC fractions by mass spectrometry. Before analysis by MS the proteins of the different fractions were separated by SDS-PAGE as depicted in Fig. 5. Table 1 lists only those proteins that were detected with a minimum of two polypeptides and a protein coverage larger than 1000. The complete protein composition of the 18 and 21 kDa FCP bands can be found in Additional file 5

Fraction number	18 kDa FCP band	21 kDa FCP band
**Fraction 2**	Lhcf1	Lhcf8
	Lhcf2	Lhcf9
	Lhcf4	
	Lhcf5	
	Lhcf6	
	Lhcf8	
	Lhcf9	
**Fraction 3**	Lhcf1	Lhcf8
	Lhcf2	Lhcf9
	Lhcf5	
	Lhcf6	
**Fraction 4**	Lhcf1	Lhcf8
	Lhcf2	Lhcf9
	Lhcf5	
	Lhcf6	
**Fraction 5**	Lhcf8	Lhcf8
	Lhcf9	Lhcf9

The analysis was extended to the complete protein composition of Fractions 1 to 4 with a special emphasis on Lhcr/Lhcx proteins and protein subunits of the PSI and PSII core complexes (Mass spectrometry analysis 2, Table 2 and Additional file 6). Fraction 5 was omitted from Mass spectrometry analysis 2 because the first analysis showed that this fraction consisted only of the Lhcf8 and Lhcf9 antenna proteins. Fraction 1 contained three protein subunits of PSII, namely the inner antenna protein CP43 (psaC), the Cytb_559_ subunit of the PSII reaction centre (psbE) and psbV, representing a subunit of the OEC, while the other fractions contained protein subunits of both PSII and PSI. Fraction 2 contained CP43 (psbC) and the second protein of the inner PSII antenna CP47 (psbB), besides the two reaction centre proteins D1 (psbA) and D2 (psbD) and the psbE and psbV subunits. Besides these PSII proteins, the smaller protein subunits psaD, psaF and psaL of PSI were detected. In Fraction 3 the PSII proteins psbA, psbC, psbD, psbE and psbV and the PSI proteins psaD, psaF and psaL were identified. The most important protein subunit of the OEC, the manganese stabilizing protein PsbO, was detected in Fractions 2 and 3 (see Additional file 6), but did not meet the criteria to be listed in Table 2, maybe because of a partial loss during solubilization. Fraction 4 contained the three PSI protein subunits psaD, psaF and psaL. In contrast to Fractions 2 and 3 the number of PSII proteins was lower and only psbC and psbE were detected in Fraction 4.

**Table 2:** Analysis of the protein composition of the FCPs and protein subunits of the PSII and PSI core complexes of the different AEC fractions by mass spectrometry. Before analysis by MS the proteins of the different fractions were separated by SDS-PAGE as depicted in Fig. 5. Table 2 lists only those proteins that were detected with a minimum of two polypeptides and a protein coverage larger than 1000. The complete protein composition of the different AEC fractions with respect to FCPs, PSI and PSII proteins can be found in the Additional file 6

Fraction number	FCP proteins	PS proteins
**Fraction 1**	Lhcx6_1	psbC
		psbE
		psbV
**Fraction 2**	Lhcf1	psbA
	Lhcf2	psbB
	Lhcf4	psbC
	Lhcf5	psbD
	Lhcf6	psbE
	Lhcf7	psbV
	Lhcf8	
	Lhcf9	psaD
		psaF
	Lhcx1	psaL
	Lhcx2	
	Lhcx5	
	Lhcr3	
**Fraction 3**	Lhcf1	psbA
	Lhcf2	psbC
	Lhcf4	psbD
	Lhcf5	psbE
	Lhcf6	psbV
	Lhcf8	
	Lhcf9	psaD
		psaF
	Lhcr3	
	Lhcr14	
**Fraction 4**	Lhcf1	psbC
	Lhcf2	psbE
	Lhcf4	
	Lhcf5	psaD
	Lhcf6	psaF
	Lhcf8	psaL
	Lhcf9	
	Lhcr1	
	Lhcr3	

Besides the PSII and PSI core proteins, FCP proteins were also identified. It should be noted that the analysis of whole gel lanes cut into 12 pieces did not distinguish between the 18 and 21 kDa bands. However, the above described separate analysis of both bands was confirmed and a few further proteins were confidently identified, i.e. Lhcf7 in Fraction 2 and Lhcf4 in Fractions 3 and 4. Furthermore, Fraction 3 contained the Lhcr3 and Lhcr14 proteins, Fraction 4 both Lhcr1 and Lhcr3 proteins, and Fraction 2 the Lhcr3 protein. With respect to the photoprotective Lhcx proteins, Lhcx1, Lhcx2 and Lhcx5 were detected in Fraction 2 and Lhcx6_1 in Fraction 1. It is interesting to note that the Lhcx proteins were not present in the 18 or 21 kDa FCP bands, but in gel pieces corresponding to an apparent molecular weight range above the 21 kDa FCP band and below the 29 kDa marker protein band (Fig. 5). Additional proteins not confidently identified are listed in Additional file 6, such as the main protein subunits of the PSI core complex, i.e. psaA and psaB, with protein scores slightly below 1000, in Fractions 2 to 4 containing the confidently identified PSI subunits psaD, psaF and psaL (Table 2). Among the proteins that do not represent PSI or PSII pigment protein complexes, the diadinoxanthin cycle enzyme diadinoxanthin de-epoxidase (DDE) should be mentioned (Additional file 6). DDE was present in Fraction 2 which was enriched in the subunits of the PSII core complex and Lhcx proteins.

## Discussion

### Assignment of the separated AEC fractions

Based on the protein determination by SDS-PAGE and mass spectrometry the AEC fractions 2 to 4 could be assigned to PSII and PSI. Although Fraction 1 contained protein subunits of PSII, the low amounts of protein, but high concentrations of pigments, in this fraction makes it unlikely that Fraction 1 consists of specific pigment protein complexes. The dominance of PSII protein subunits in Fraction 2 argues for the presence of a high amount of PSII core complexes in this fraction. Fraction 3 contained protein subunits of both PSII and PSI and seems to represent a fraction with a mixed population of PSII and PSI core complexes. Fraction 4, on the other hand, was characterized by a lower number of PSII proteins compared to Fractions 2 and 3 but still contained the three PSI subunits which were typically observed in the present study. This argues for a higher concentration of PSI core complexes in Fraction 4. Fraction 5, which represented the main peak of the AEC chromatogram, was characterized by a strong enrichment of FCP proteins and thus most likely represents the peripheral FCP complexes of *T. pseudonana*. The enrichment of PSII core complexes in Fraction 2 and PSI in Fraction 4 was in line with the spectroscopic characterization of these fractions. Fraction 2 contained Chl a molecules absorbing at shorter wavelengths in the red part of the spectrum which are typical for PSII. Fraction 4, on the other hand, was characterized by the presence of longer-wavelength absorbing and fluorescence emitting Chl a molecules typical for PSI. Fraction 5 contained Chl a molecules which were absorbing at shorter wavelengths in the red part of the spectrum which is in line with the presence of FCP complexes in this fraction. The high fluorescence emission of Fraction 5 in the long-wavelength region is most likely caused by a strong aggregation of the FCPs by the high salt concentration needed for elution like in the experiments of Schaller et al. [30] who used Mg^2+^ ions to aggregate the FCP complexes. In some cases a short wavelength emission was observed for Fraction 5. In this case it is reasonable to believe that the FCP complexes in Fraction 5 showed a weaker aggregation. Differences in the aggregation state of the FCP complexes in Fraction 5 may have been caused by slight differences in the solubilisation conditions of the thylakoid membranes, which, in general, could not be isolated with such a high reproducibility as e.g. spinach thylakoids. Fraction 5 showed high Fx per Chl a and Fx per DD ratios which is typical for FCP complexes with a primary light-harvesting function. Although Fraction 5 contained the largest part of the FCP complexes of *T. pseudonana*, FCP complexes were also present in Fractions 2 to 4. However, the higher β-carotene concentrations of Fractions 2 to 4 indicate that the PSI and PSII core complexes and not the FCP complexes were enriched in these fractions. The presence of FCP complexes in the isolated PSII core complexes observed in the present study is in line with studies of Nagao et al. [26, 28]. In these studies thylakoid membranes of the centric diatom *C. gracilis* were solubilized with Triton X-100 and oxygen-evolving PSII core complexes were isolated by differential centrifugation [26]. These FCP containing PSII preparations could then be further purified by anion exchange chromatography [28]. Like in our present AEC separation Ikeda et al. [17, 18] isolated PSI core complexes with associated FCP complexes from the centric diatoms *C. gracilis* and *T. pseudonana* with the help of sucrose gradient centrifugation and size exclusion chromatography [17] or sucrose gradient centrifugation in combination with AEC [18]. The isolation procedures led to the purification of PSI core complexes with two different FCP complexes which were termed FCPI-1 and FCPI-2. FCPI-2 seems to be tightly associated with the PSI core complex while FCPI-1 is lost after a more severe detergent treatment. Ikeda et al. [18] proposed that the FCPI-2 complex mediates the excitation energy transfer between the more peripheral FCPI-1 and the PSI core.

According to the recent data of Gundermann et al. [16] who purified the FCP complexes of the centric diatom *C. meneghiniana* with a combination of AEC and sucrose density gradient centrifugation it is possible that during the AEC separation described in the present study a co-elution of FCPa complexes and PSII and PSI core complexes has taken place. The AEC elution profile presented by Gundermann et al. [16] shows a pronounced peak at high salt concentrations which has been assigned to the FCPb complex. Additional smaller peaks were eluted from the column at lower salt concentrations and have been characterized as different subtypes of the so called FCPa complexes. While the FCPb peak at high salt concentrations most likely corresponds to Fraction 5 of the present AEC separation the smaller FCPa peaks exhibit retention times which are comparable with the retention times of the PSII and PSI fractions, i.e. Fractions 2 to 4, of our protein separation. The possible co-elution of the FCPa complexes and the PSII/PSI fractions makes it difficult to decide if the FCPs found in the present PSII and PSI fractions represent antenna proteins which are tightly associated with the photosystem core complexes or if these proteins are subunits of the different FCPa complexes of *T. pseudonana*.

Separation of the pigment protein complexes of *T. pseudonana* with the method presented in this study was compared to the separation of the photosynthetic pigment proteins of spinach (Additional file 2A). Separation of the spinach pigment proteins led to the appearance of one major and several minor peaks. The major peak, which eluted at 12–15 mL, showed pronounced Chl a and Chl b maxima in the blue and red part of the spectrum and could be unequivocally assigned to the LHCII (Additional file 3). The short retention time of the LHCII indicates that the major light-harvesting complex of higher plants exhibits a rather low negative net charge and thus could be eluted from the AEC column with low salt concentrations. The peripheral FCP of *T. pseudonana*, on the other hand, was characterized by the longest retention time of the separated diatom pigment protein complexes and only eluted at high concentrations of NaCl, which indicates a high negative charge of the FCP complexes. Comparing the LHCII and the peripheral FCP complexes it is possible that the exposed regions of the proteins, which interact with the positively charged matrix of the AEC column, show differences in their negative charge. It is also possible that differences in the oligomerization state of the light-harvesting complexes lead to the different negative net charges and thus the different retention times. The LHCII of higher plants is usually isolated as trimeric LHCII. Higher oligomeric states of FCP complexes are typical for the centric diatoms like *C. meneghiniana* or the diatom used in the present study, *T. pseudonana*. In these algae the FCPb complexes, which represent the last protein fraction in the purification of FCP complexes by AEC [16], and thus are comparable to Fraction 5 of the present separation, seem to be composed of FCP nonamers while FCPa complexes show a trimeric structure [3, 13, 27]. The increased negative surface charge of FCPs may be seen in conjunction with the high concentration of the negatively charged lipid SQDG in the thylakoid membranes of diatoms [22]. Pronounced repulsion between FCPs and SQDG may lead to the separation of PSI into SQDG enriched outer thylakoid membrane regions and PSII and the peripheral FCP into the inner membrane lamellae composed of mainly MGDG. Such a separation of the photosystem has been proposed by Lepetit et al. [22] and has recently been supported by the data of Bina et al. [4] and Flori et al. [10].

### Applicability of the present AEC separation method

The AEC method presented in this study allows the pre-purification of PSI and PSII core and FCP complexes of the centric diatom *T. pseudonana*. It was also tested for the separation of the pigment proteins of the well-characterized centric diatom *C. meneghiniana*. These analyses yielded a comparable fractionation of the solubilized thylakoid membranes (see Additional file 2B). The isolated pigment protein complexes can serve as starting material for the final purification of the respective complexes using a separate protein purification method such as size exclusion chromatography or sucrose density gradient centrifugation. The partial purification, i.e. the isolation of PSI and PSII core complex fractions which contain FCP complexes, makes it possible to differentiate between FCP complexes which are more closely associated with the PSI and PSII core complexes and FCP complexes which build-up the peripheral antenna complexes, providing that the occurrence of FCPs in the PSI and PSII fractions does not represent a co-elution of FCP-A complexes and PS core complexes.

### Heterogeneity of FCPs

Fraction 5 of the present AEC separation contained the peripheral FCP complexes which were not associated with the PSI and PSII core complexes. The peripheral FCP complexes were dominated by the presence of the 21 kDa protein band and the 18 kDa FCP band was only detected in low concentration. According to the analysis by mass spectrometry the 21 kDa band was exclusively composed of both the Lhcf8 and Lhcf9 proteins. Interestingly, Lhcf8 and Lhcf9 were also found in the 18 kDa band but, based on the data of analysis 1 of the present study, not in all fractions of the AEC. Additional Lhc proteins were not detected in the peripheral FCP. According to the AEC separation presented by Gundermann et al. [16] Fraction 5 corresponds to the FCPb complexes of *C. meneghiniana*. Gundermann et al. [16] detected Fcp5/Lhcf3 as the most prominent Lhc protein in the FCPb complexes. Lhcf3 was accompanied by low concentrations of Lhcf1 and Lhcf4/Lhcf6. The latter, however, was only found in the FCPb2 complex. Taking into account that in *T. pseudonana* the similar Lhcf3, Lhcf8 and Lhcf9 genes code for an identical protein, the data of the present study on the protein composition of the peripheral FCP complexes are in agreement with the data of Gundermann et al. [16] concerning the FCPb. In addition to the Lhcf proteins, Gundermann et al. [16] observed the presence of the Lhcx1 and Lhcx6_1 proteins in the FCPb which was purified from high light grown cultures. These proteins were not detected in the present study as components of the peripheral FCP. The occurrence of the Lhcf8 protein in the 21 kDa FCP band is in line with the data published by Nagao et al. [27] who detected the Lhcf8 protein in the 21 kDa bands of the oligomeric and trimeric FCP of *T. pseudonana*. The Lhcf9 protein, which was detected as an additional component of the 21 kDa band in the present study, was not observed by Nagao et al. [27]. However, this protein is likely identical to the Lhcf8 gene product. The oligomeric FCP of *T. pseudonana*, which was purified by Nagao et al. [27] by clear-native PAGE, was characterized by the single presence of the 21 kDa FCP band. It is thus comparable to the peripheral FCP complexes isolated in the present study which were also dominated by the 21 kDa band. The findings of the present study are also in line with recent observations by Calvaruso et al. [6] that the peripheral FCPb complex of *T. pseudonana* consists of the Lhcf8/Lhcf9 proteins. Like the 21 kDa band of Fraction 5 the 21 kDa bands of the other AEC fractions contained only Lhcf8 and Lhcf9. Like the 21 kDa band the 18 kDa band showed a comparable protein composition in the different fractions with the exception of Lhcf8 and Lhcf9 which, according to analysis 1, were only found in the 18 kDa band of Fractions 2 and 5. Lhcf1, Lhcf2, Lhcf5, and Lhcf6 on the other hand, were found in all fractions and thus seem to represent the main Lhcf proteins of the 18 kDa band. While in the first MS analysis performed in the present study Lhcf4 was only detected in the 18 kDa band of Fraction 2, the second MS analysis indicated the presence of the Lhcf4 protein in the 18 kDa band of Fractions 2 to 4. The presence of Lhcf5 in the 18 kDa FCP band is in line with the data of Nagao et al. [27] who observed two FCP bands in the 18 kDa region of trimeric FCP complexes of *T. pseudonana*. According to their mass spectrometric analysis the major 18 kDa band contained Lhcf5 and additionally Lhcf1 and Lhcf4. In the present study both Lhcf1 and Lhcf4 were also detected in the 18 kDa band of Fractions 2 to 4. The minor 18 kDa band in the study of Nagao et al. [27] was characterized by the additional presence of Lhcf6, Lhcf7, and Lhcf11. Lhcf6 represented a component of Fractions 2 to 4 of the present AEC separation, whereas Lhcf7 was only detected in the second analysis by mass spectrometry and only occurred in Fraction 2. Lhcf11, however, was not observed in the respective fractions of the present study. The absence of Lhcf11 is most probably explained by the fact that in the present protein separations by SDS-PAGE a minor 18 kDa FCP band was not resolved. The presence of Lhcf4 in the 18 kDA band of Fraction 2, which seems to be enriched in PSII, is in line with the recent isolation of a PSII-FCP supercomplex of *C. gracilis* [35]. Based on the structural data it was proposed that the FCP-E monomer, which is rather tightly associated with the PSII core complex and mediates the interaction of one of the FCP-A tetramers with the core, represents an Lhcf4-like subunit. Interestingly, the FCP-D monomer, which is also involved in the interaction of the peripheral FCP-A with the PSII core complex, seems to be an Lhca-related LHC protein.

Taking into account that a co-elution of the PSII and PSI core complexes and the FCPa complexes might have taken place in the AEC separation of the present study a comparison to the protein composition of the different FCPa complexes published by Gundermann et al. [16] seems valuable. In the present study Lhcf1, Lhcf2, Lhcf5, and Lhcf6, with an additional presence of Lhcf8 and Lhcf9, seemed to represent the main proteins of AEC fractions 2 to 4, which would correspond to the different FCPa complexes. Gundermann et al. [16] observed that the FCPa complexes of *C. meneghiniana* were dominated by the Lhcf1, Lhcf4/Lhcf6 and Lhcf3 proteins. According to their analysis by mass spectrometry, FCPa1, FCPa3 and FCPa4 contain Lhcf1 as the main Lhcf protein whereas FCPa2 is characterized by a high concentration of Lhcf4/Lhcf6.

Additional FCP proteins that were detected by Gundermann et al. [16] as constituents of the FCPa complexes were the Lhcx1 and Lhcx6_1 protein. In the present study four different Lhcx proteins were found, namely Lhcx1, Lhcx2, Lhcx5 and Lhcx6_1. The Lhcx1, Lhcx2 and Lhcx5 proteins were observed in Fraction 2 of the AEC separation, which according to its protein composition, represents a fraction enriched in PSII core complexes. Lhcx6_1 was a constituent of Fraction 1 which, due to its high concentration of DD, Dt and Fx, has to be regarded as a mixed protein and free pigment fraction.

Fractions 3 and 4 did not contain Lhcx proteins but, in addition to the Lhcf proteins, were characterized by the presence of two Lhcr proteins in each fraction. While Fraction 3 contained Lhcr3 and Lhcr14, Lhcr1 and Lhcr3 were found in Fraction 4. In addition, Lhcr3 was detected in Fraction 2. The presence of Lhcr proteins in the AEC fractions containing PSI core complex proteins is in line with data from the literature which describe the Lhcr proteins as PSI-specific antenna proteins [13, 18]. While in the present study Lhcr1, Lhcr3 and Lhcr14 were detected, Grouneva et al. [13] observed Lhcr1, Lhcr3, Lhcr4, Lhcr7, Lhcr10, Lhcr11 and Lhcr14 in their analysis of the thylakoid proteome of *T. pseudonana*. The analysis of PSI-FCPI complexes of *T. pseudonana* isolated by a combination of sucrose gradient centrifugation and AEC [18] showed the presence of Lhcr1, Lhcr3, Lhcr4, Lhcr10, Lhcr13 and Lhcr14 as PSI antenna proteins.

### Assignment of other important proteins

Fraction 2 of the present AEC separation, which is enriched in PSII core complexes, contains another interesting protein, namely the DD de-epoxidase (DDE). DDE is the enzyme which catalyses the forward reaction of the xanthophyll cycle of diatoms, the de-epoxidation of DD to Dt (for a review on xanthophyll cycles and NPQ see [11]). Dt is one of the components responsible for the process of NPQ. Another important factor for NPQ is the presence of Lhcx proteins which also occur in the PSII-containing Fraction 2 of the AEC gradient. Dt and Lhcx proteins are thought to play a role in the quenching site Q2 which is located in the vicinity of the PSII core complex and, together with quenching site Q1, provides protection of PSII against damages caused by excessive excitation energy.

## Conclusion

The results of the present study add a little bit to the big puzzle of the diatom antenna system. They corroborate existing data like the observation of a PSI-specific antenna complex in diatoms composed of Lhcr proteins. They complement other data, like e.g. on the protein composition of the 21 kDa FCP band or the Lhcf composition of FCPa and FCPb complexes. Here the data indicate that the Lhcf composition of FCPa complexes of the centric diatoms *T. pseudonana* and *C. meneghiniana* shows similarities but also differences. The present data also provide some interesting new information like the presence of the enzyme DDE in the PSII fraction which might be seen in connection with the PSII-specific Lhcx proteins and the role of the xanthophyll cycle and the Lhcx proteins in the establishment of NPQ. In addition, the present data indicate that the net charge of the main LHCs of higher plants and diatoms is significantly different which might result from the different higher order LHC structures. The high negative charge of the main FCP fraction may be responsible for the confinement of these FCP complexes in the inner membranes of the typical stacks of three thylakoid membranes of diatoms as proposed by the latest models. Further experiments on the native structure of diatom photosynthetic protein complexes should also address the role of membrane lipids, especially the significance of the negatively charged SQDG, which is found in high concentrations in diatom thylakoid membranes.

## Methods

### Plant material

*T. pseudonana* cultures (Culture Collection of Algae and Protozoa, strain CCAP 1085/12) were grown in F/2 medium according to Guillard [14] with a double concentration of silicate and a 50% reduced salt content. The cells were cultivated as sterile airlift cultures at a temperature of 20 °C. The growth light conditions consisted of a light/dark regime of 14/10 h and an incident light intensity of 40 μmol photons m^− 2^ s^− 1^. For the preparation of thylakoids and pigment protein complexes cells were harvested during the logarithmic growth phase.

### Isolation of thylakoids and pigment protein complexes

Thylakoid membranes of *T. pseudonana* were isolated according to the method of Lepetit et al. [24] and stored at − 80 °C until further use. The chlorophyll concentration of the isolated thylakoid membranes was determined in 90% acetone according to Jeffrey and Humphrey [19].

For the preparation of pigment protein complexes thylakoid membranes were solubilized in a medium consisting of 2 mM KCl, 5 mM EDTA and 10 mM MES (pH 6.5, room temperature (RT)). The Chl concentration during solubilization was adjusted to 1 mg mL^− 1^ and the detergent n-dodecyl β-D-maltoside (β-DM) was used with a β-DM /Chl ratio of 20, corresponding to a β-DM concentration of around 40 mM. The solubilization was carried out on ice in the dark for 20 min with a gentle stirring of the sample. After solubilization un-solubilized thylakoid fragments were removed by centrifugation with 21.380 g for 10 min at 4 °C (Allegra 64R, Beckman Coulter, USA). The supernatant containing the solubilized pigment protein complexes was then separated by AEC using a NGC Chromatography System (BioRad, USA) equipped with a MonoQ 5/50 GL column (Sigma-Aldrich, USA). Elution of the pigment protein complexes was achieved by a gradient with seven steps from Eluent A (30 mM KCl, 0.03% β-DM, 20 mM HEPES, pH 7.5, RT) to Eluent B (500 mM KCl, 0.03% β-DM, 20 mM HEPES, pH 7.5, RT) at 10 °C in the dark with a flow rate of 1 mL min^− 1^ (see Additional file 4). Proteins were detected by recording the absorbance at 280 nm. During the AEC separation fractions with a volume of 1 mL were collected. After the AEC run the fractions containing the separated pigment protein complexes were collected, pooled and concentrated by ultrafiltration tubes with a pore size of 10 kDa (Amicon Ultra-4, Merck Millipore, USA) and centrifugation with 3.000 g at 4 °C (Thermo Scientific 400R, USA).

### Absorption and 77 K fluorescence spectroscopy

Absorption spectra of the different AEC fractions were recorded in a Specord M250 photometer (Zeiss, Germany) in a wavelength range from 350 to 750 nm with a bandpass setting of 1 nm. 77 K fluorescence spectroscopy of the AEC fractions was performed in a Fluoromax 4P fluorometer (Horiba Jobin Yvon, France). The fractions were adjusted to an absorbance of 0.1 of the Chl a maximum in the red part of the spectrum and then further diluted with glycerol until a final glycerol concentration of 60% was obtained. Fluorescence emission spectra were recorded in a wavelength range from 600 to 800 nm with an excitation wavelength of 440 nm. The bandwidths of the emission and excitation light were adjusted to 2 and 5 nm, respectively. For the fluorescence excitation spectra a wavelength range from 400 to 550 nm was chosen and the emission wavelengths were set to the respective fluorescence emission maxima of the different AEC fractions. For the excitation spectra the bandwidths for the emission and excitation light were set 5 nm and 2 nm, respectively. The device was calibrated following the instructions of the manufacturer. Excitation spectra were corrected automatically against the spectrum of the light source.

### Pigment extraction and determination by HPLC

Total pigments were extracted by adding pigment extraction medium (CHCl_3_:MeOH:NH_3_, in the ratio of 1:2:0.004, v/v) to an equal volume of the different AEC fractions. After vortexing and a short centrifugation with 16.000 g for 2.5 min (Sigma 1-14 K, Sigma, Germany) a clear separation between the aqueous and the organic phase could be observed. The lower organic phase containing the pigments was collected, dried under a gentle stream of nitrogen and stored at − 20 °C until pigment analysis by HPLC was performed.

The dried pigment extracts of the different AEC fractions were dissolved in a medium consisting of 90% methanol/0.2 M ammonium acetate (9:1, v/v) and 10% ethyl acetate. The pigments were then analysed on a Waters 600-MS chromatography system with a Waters 996 photodiode array detector (Waters, USA) equipped with a Nucleosil ET 250/8/4, 300–5, C-18 column (Macherey & Nagel, Germany). The eluents and gradient program used for the separation were derived from a method first described by Kraay et al. [20]. After separation the pigments were quantified according to Lohr and Wilhelm [25].

### Protein analysis by SDS-PAGE

AEC fractions were analysed by SDS-PAGE according to Laemmli [21] using a Mini-PROTEAN Tetra Cell system (BioRad, USA). Stacking and separation gels were prepared with acrylamide concentrations of 4 and 15%, respectively. Samples were loaded on the gel with a Chl content of 0.5 μg. The proteins were stained with colloidal Coomassie Brilliant Blue G-250 solution according to Dyballa and Metzger [8].

### Protein analysis by mass spectrometry

Two protein gels were analyzed by mass spectrometry. For the first analysis the 18 and 21 kDa bands of the AEC fractions, representing the FCPs, were excised with the ExQuest™ Spot Cutter (Bio-Rad Laboratories, Hercules, California, USA) and transferred to different 0.5-mL reaction tubes (Eppendorf Vertrieb Deutschland GmbH, Hamburg, Germany). For the second analysis, the whole gel lane of an AEC fraction was manually cut into 12 gel pieces of equal size and transferred to different 0.5-mL reaction tubes. Gel pieces excised with the ExQuest™ Spot Cutter were washed three times (5 min, 100 μL 30% (v/v) acetonitrile in 50 mmol/L ammonium bicarbonate) and dehydrated with acetonitrile (5 min, 100 μL). The same protocol was applied to the hand-cut gel pieces, but the applied volumes were doubled. Gel pieces were rehydrated with 2 μL trypsin solution (Serva Electrophoresis GmbH, Heidelberg, Germany, 50 ng/μL in 3 mmol/L aqueous ammonium bicarbonate) and 18 μL (for hand-cut 38 μL) 3 mmol/l aqueous ammonium bicarbonate and incubated at 37 °C. After 4 h the supernatant of each sample was transferred to a new 0.5-mL reaction tube. Remaining gels pieces were washed with 60% (v/v) aqueous acetonitrile containing 0.1% formic acid and acetonitrile (20 μL per tube, 40 μL per tube for hand-cut pieces, 5 min, RT). Supernatants were transferred to the reaction tube containing the first supernatant and were dried in a vacuum concentrator 5301 (Eppendorf Vertrieb Deutschland GmbH, Hamburg, Germany) for 1 h, 60 °C.

NanoRP-UPLC-ESI-QTOF-MS/MS was performed as described [9]. Protein Lynx Global server (PLGS, version 3.0.3) was used for data analysis. The following processing and workflow parameters were used. Apex3D relied on 120 counts for LE data and 30 counts for HE data. Database Uniprot “*Thalassiosira pseudonana*” (54.905 sequences, downloaded 14th November 2018), two missed cleavage site, trypsin_P as “digester reagent” and methionine oxidation as variable modification.

## Supplementary information

**Additional file 1.** Pigment composition of the different AEC fractions (depicted as mM pigment M^− 1^ Chl a). Mean values of three independent preparations with the respective standard deviations are depicted. A: Chl c content of the AEC fractions, B: fucoxanthin content of the AEC fractions, C: diadinoxanthin content of the fractions, D: diatoxanthin content of the fractions and E: β-carotene content of the AEC fractions.

**Additional file 2 **Comparison of the elution profiles of the pigment protein complexes of *T. pseudonana* and spinach (A) and *T. pseudonana* and the well-studied centric diatom *C. meneghinina* (B) separated by anion exchange chromatography (AEC). Before the separation isolated spinach thylakoids or the thylakoids of the two diatoms were solubilized with a β-DM per Chl ratio of 20. Asterisk indicates the fraction of spinach of which the absorption spectrum is shown in Additional file 3.

**Additional file 3.** Absorption spectrum of the major fraction of the separated pigment protein complexes of spinach (marked with an asterisk in Additional file 2). The absorption spectrum was normalized to the Q_Y_ band of Chl a. For the measurements the Chl concentration of the isolated pigment protein complexes was adjusted in such a way that the absorption in the blue part of the spectrum did not exceed absorption values of 1.

**Additional file 4 **Elution profile used for the separation of the pigment protein complexes of *T. pseudonana* based on column volume (CV, 0.98 mL for MonoQ 5/50 GL). Negative CV indicates elution before onset of the gradient, including column equilibration, sample application and column wash. The step gradient starts at CV = 0 corresponding to an elution volume of 7.84 mL. Elution volume corresponds to the x-axis in Fig. 1 and Additional file 2. Eluent A consisted of 30 mM KCl, 0.03% β-DM, 20 mM HEPES, pH 7.5, Eluent B of 500 mM KCl, 0.03% β-DM, 20 mM HEPES, pH 7.5. Flow rate was set to 1 mL min^− 1^. For further information see the Methods section.

**Additional file 5.** Detailed information for the analysis of the 18 and 21 kDa FCP bands of the different AEC fractions by mass spectrometry. In contrast to Table 1 in the Results section, which only depicts the FCP proteins that were determined with a minimum of two polypeptides and a protein coverage larger than 1000, Additional file 5 lists all the FCP proteins that were detected in the MS analysis. In addition, this file provides the most important PLGS protein and peptide data for all detected proteins.

**Additional file 6.** Detailed information for the analysis of the protein composition of the FCPs and protein subunits of the PSII and PSI core complexes of the different AEC fractions by mass spectrometry. In contrast to Table 2 in the Results section, which only depicts the FCP, PSI and PSII proteins that were determined with a minimum of two polypeptides and a protein coverage larger than 1000, Additional file 6 lists all the FCP, PSI and PSII proteins that were detected in the MS analysis. In addition, this table provides the most important PLGS protein and peptide data for all detected proteins.

**Additional file 7 **Protein composition of the five AEC fractions determined by SDS-PAGE. Proteins were stained with colloidal Coomassie Brilliant Blue. M: molecular weight markers, n.d.: AEC fraction that was not further analysed in the present study, E.coli: protein extract of *Escherichia coli* that was added as a reference for the MS analysis, thy: thylakoid proteins of *T. pseudonana*. Additional file 7A depicts the original SDS-gel from which lanes 2 to 5 of Fig. 5 were derived, Additional file 7B shows the gel which was used for the depiction of lane 1 in Fig. 5.

**Additional file 8.** 77 K fluorescence emission spectra of Fraction 5 of two different, independent AEC fractionations. The spectra were normalized to the fluorescence emission maximum of the Chl a fluorescence. For further measurement details see the Methods section and the legend of Fig. 3 of the main text.

## Data Availability

The datasets used and/or analysed during the current study are available from the corresponding author on reasonable request.

## Abbreviations

AEC: Anion exchange chromatography
Car: β-Carotene
Chl: Chlorophyll
DD: Diadinoxanthin
DDE: Diadinoxanthin de-epoxidase
β-DM: n-dodecyl β-D-maltoside
Dt: Diatoxanthin
FCP: Fucoxanthin chlorophyll protein
FCPo: FCP in oligomeric state
Fx: Fucoxanthin
LHC: Light-harvesting complex
PSMGDG: Monogalactosyldiacylglycerol
MS: Mass spectrometry
NPQ: Non-photochemical quenching
OEC: Oxygen evolving complex
PSII: Photosystem II
PSI: Photosystem I
SQDG: Sulfoquinovosyldiacylglycerol
