# Supplementary figures and images for: Pre-purification of diatom pigment protein complexes provides insight into the heterogeneity of FCP complexes
Source: BMC Plant Biol. 2020 Oct 6;20:456. doi: 10.1186/s12870-020-02668-x (PMC7539453; doi:10.1186/s12870-020-02668-x)

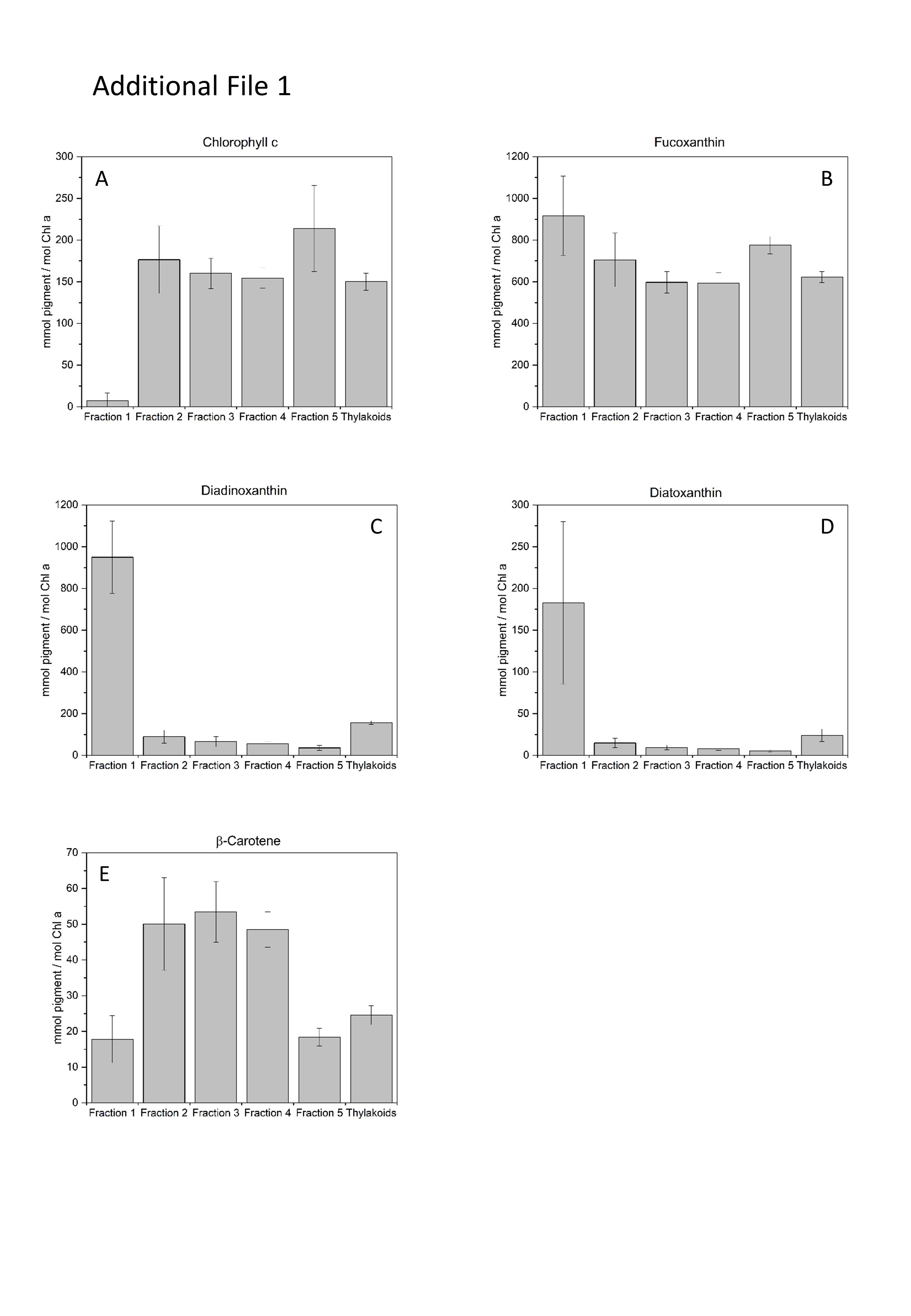

Supplement: Supplementary file 1 — Additional file 1. Pigment composition of the different AEC fractions (depicted as mM pigment M− 1 Chl a). Mean values of three independent preparations with the respective standard deviations are depicted. A: Chl c content of the AEC fractions, B: fucoxanthin content of the AEC fractions, C: diadinoxanthin content of the fractions, D: diatoxanthin content of the fractions and E: β-carotene content of the AEC fractions. [file 12870_2020_2668_MOESM1_ESM.png]

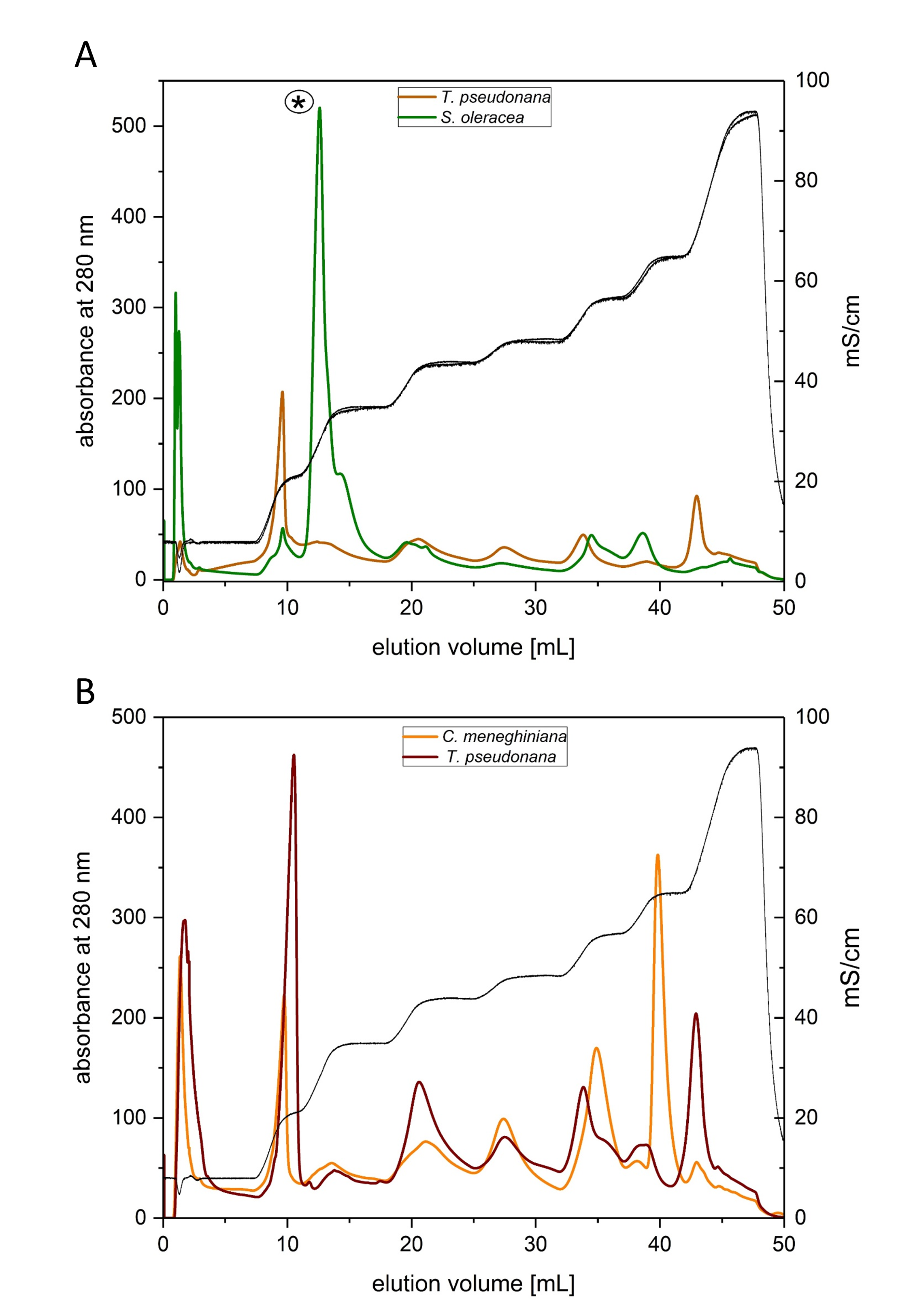

Supplement: Supplementary file 2 — Additional file 2 Comparison of the elution profiles of the pigment protein complexes of T. pseudonana and spinach (A) and T. pseudonana and the well-studied centric diatom C. meneghinina (B) separated by anion exchange chromatography (AEC). Before the separation isolated spinach thylakoids or the thylakoids of the two diatoms were solubilized with a β-DM per Chl ratio of 20. Asterisk indicates the fraction of spinach of which the absorption spectrum is shown in Additional file 3. [file 12870_2020_2668_MOESM2_ESM.png]

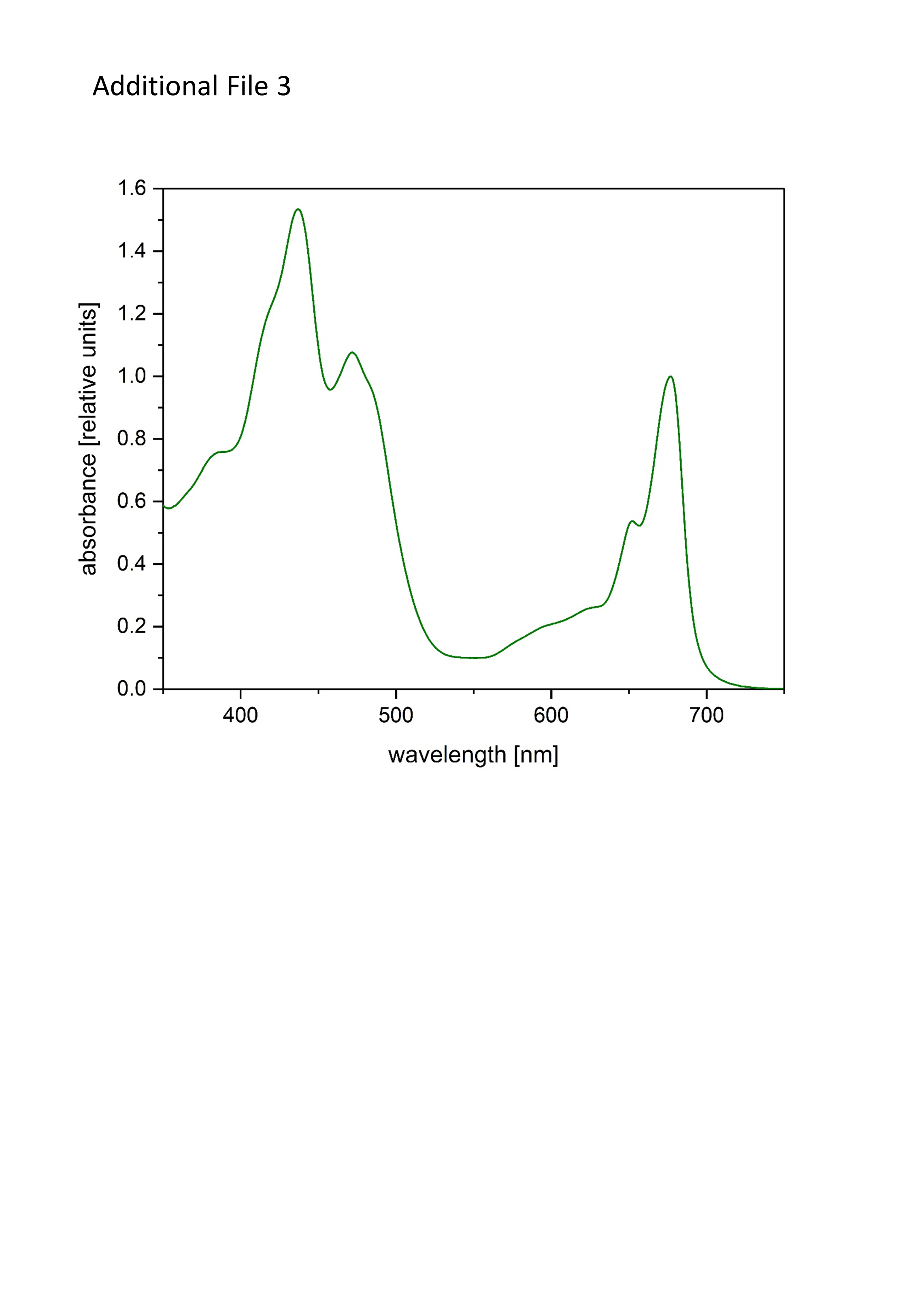

Supplement: Supplementary file 3 — Additional file 3. Absorption spectrum of the major fraction of the separated pigment protein complexes of spinach (marked with an asterisk in Additional file 2). The absorption spectrum was normalized to the QY band of Chl a. For the measurements the Chl concentration of the isolated pigment protein complexes was adjusted in such a way that the absorption in the blue part of the spectrum did not exceed absorption values of 1. [file 12870_2020_2668_MOESM3_ESM.png]

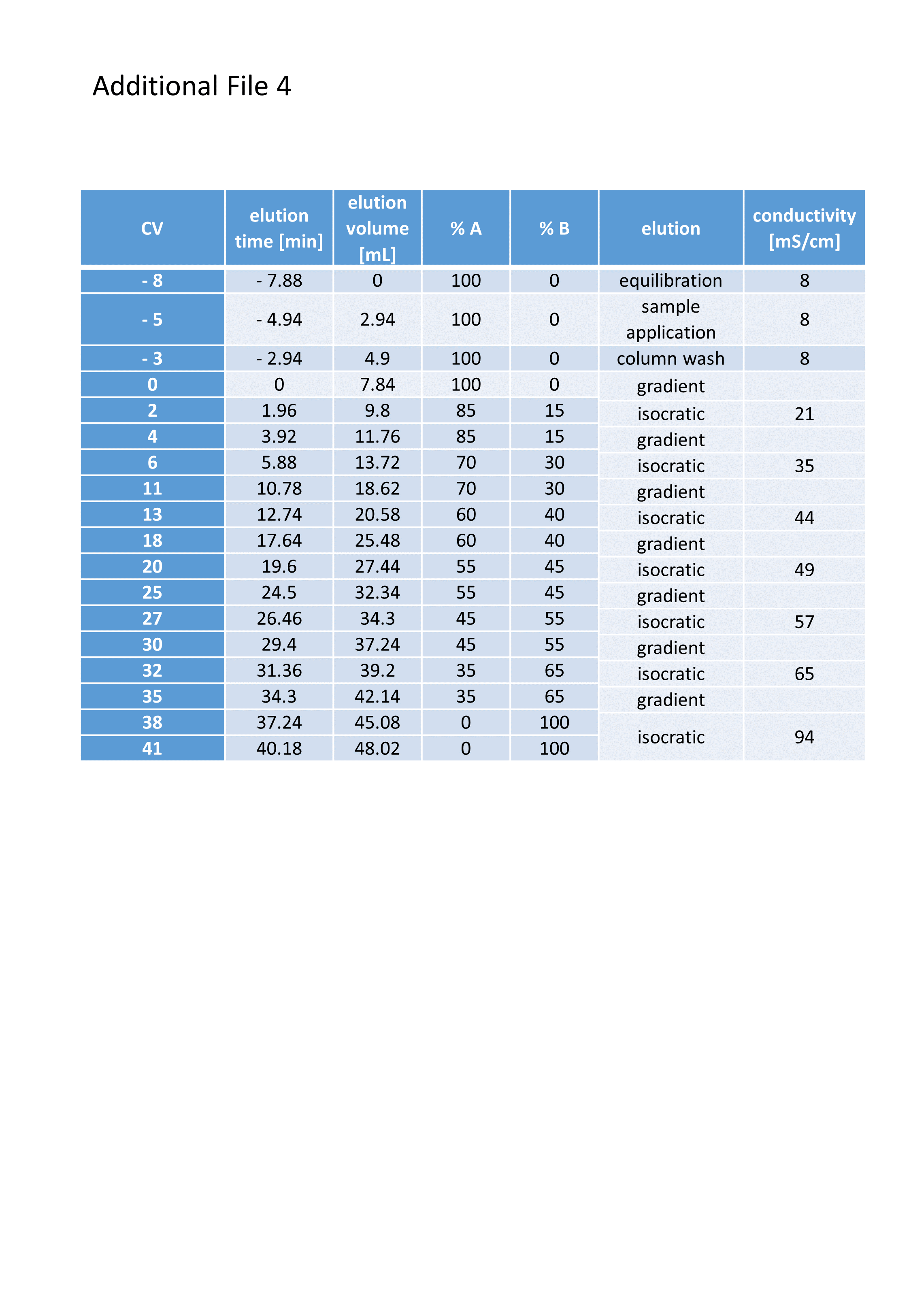

Supplement: Supplementary file 4 — Additional file 4 Elution profile used for the separation of the pigment protein complexes of T. pseudonana based on column volume (CV, 0.98 mL for MonoQ 5/50 GL). Negative CV indicates elution before onset of the gradient, including column equilibration, sample application and column wash. The step gradient starts at CV = 0 corresponding to an elution volume of 7.84 mL. Elution volume corresponds to the x-axis in Fig. 1 and Additional file 2. Eluent A consisted of 30 mM KCl, 0.03% β-DM, 20 mM HEPES, pH 7.5, Eluent B of 500 mM KCl, 0.03% β-DM, 20 mM HEPES, pH 7.5. Flow rate was set to 1 mL min− 1. For further information see the Methods section. [file 12870_2020_2668_MOESM4_ESM.png]

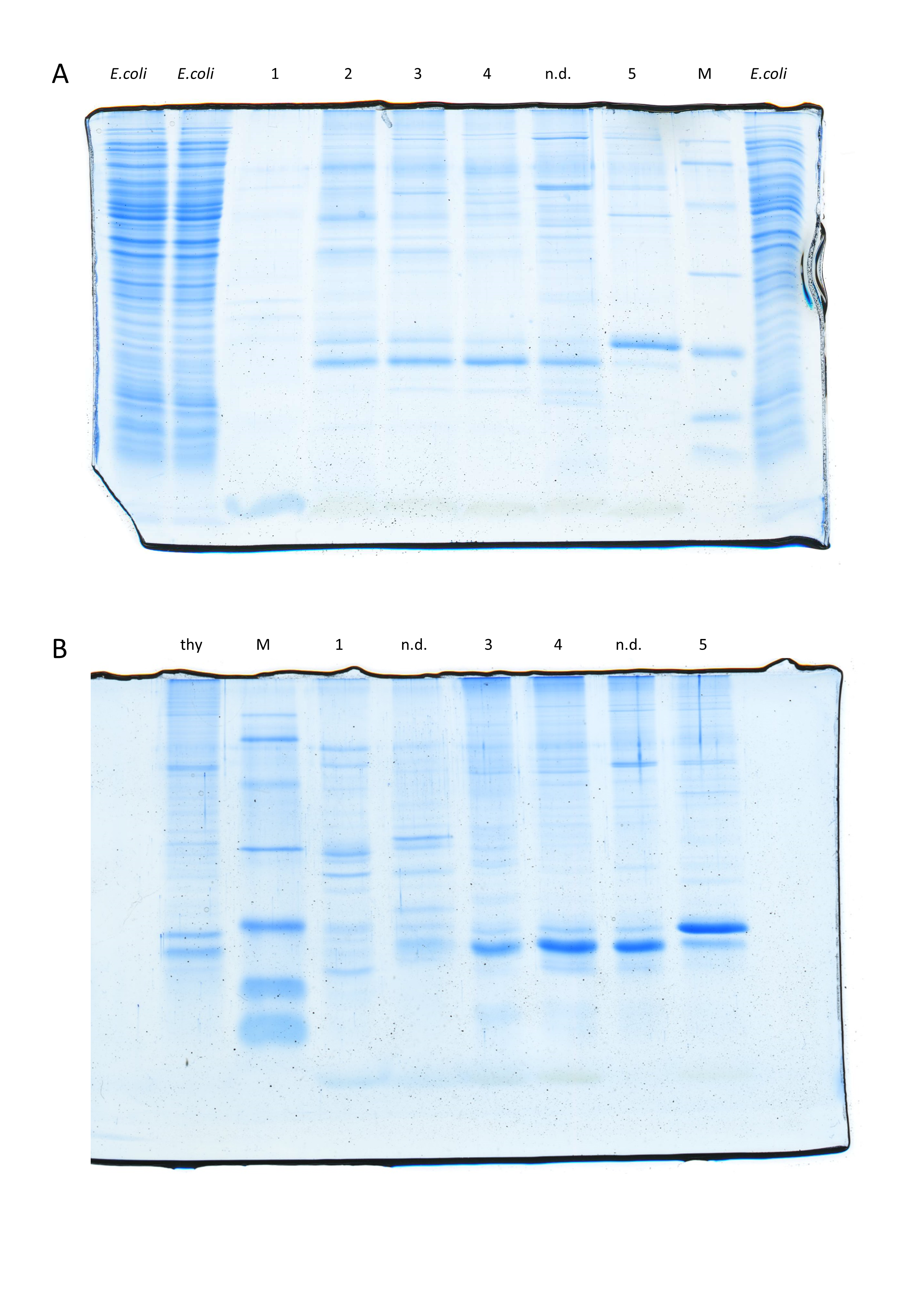

Supplement: Supplementary file 7 — Additional file 7 Protein composition of the five AEC fractions determined by SDS-PAGE. Proteins were stained with colloidal Coomassie Brilliant Blue. M: molecular weight markers, n.d.: AEC fraction that was not further analysed in the present study, E.coli: protein extract of Escherichia coli that was added as a reference for the MS analysis, thy: thylakoid proteins of T. pseudonana. Additional file 7A depicts the original SDS-gel from which lanes 2 to 5 of Fig. 5 were derived, Additional file 7B shows the gel which was used for the depiction of lane 1 in Fig. 5. [file 12870_2020_2668_MOESM7_ESM.png]

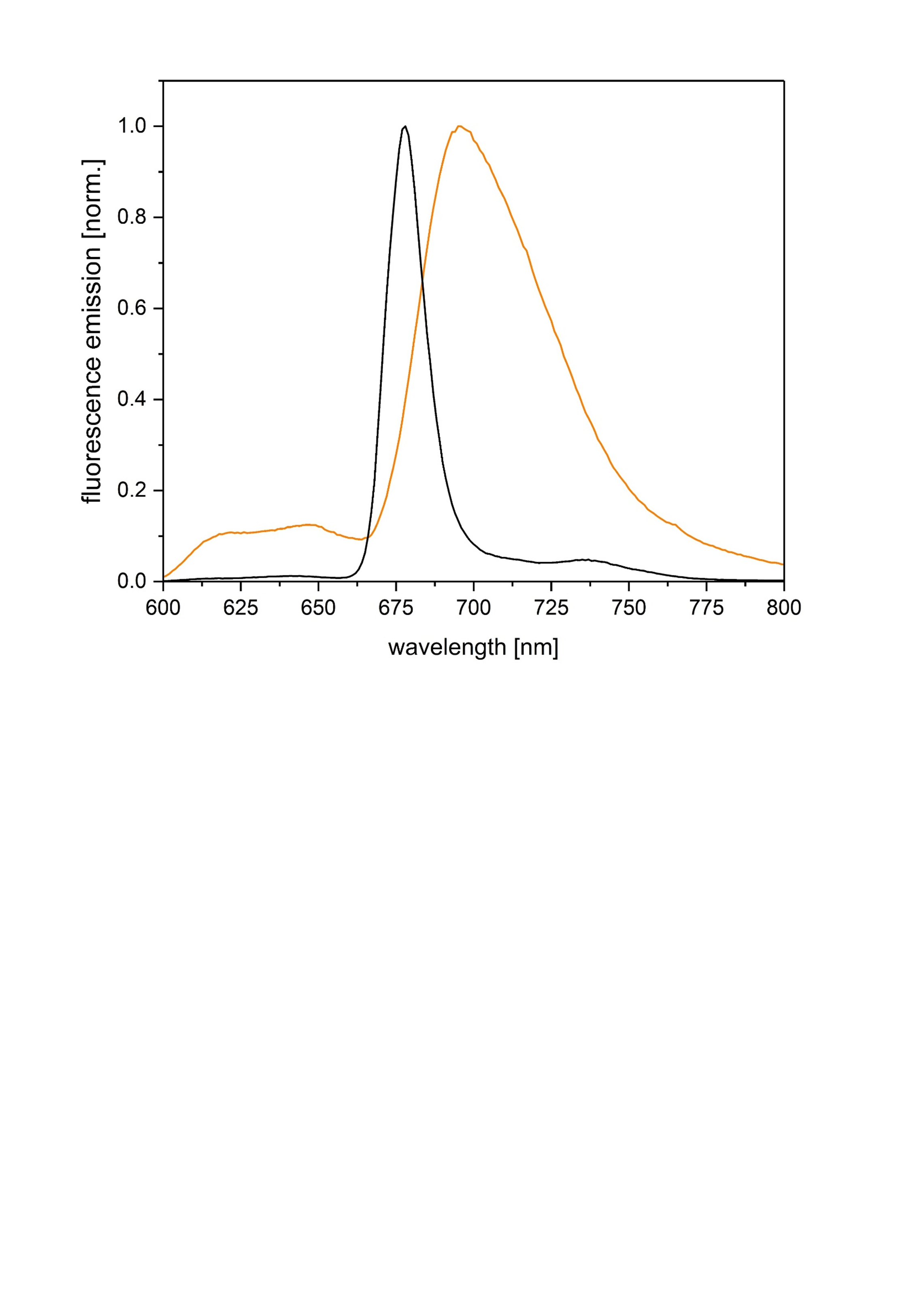

Supplement: Supplementary file 8 — Additional file 8. 77 K fluorescence emission spectra of Fraction 5 of two different, independent AEC fractionations. The spectra were normalized to the fluorescence emission maximum of the Chl a fluorescence. For further measurement details see the Methods section and the legend of Fig. 3 of the main text. [file 12870_2020_2668_MOESM8_ESM.png]
